# *Taenia ovis* in Small Ruminants in Iran: Prevalence, Pathology, and Economic Loss

**DOI:** 10.3390/vetsci7010034

**Published:** 2020-03-20

**Authors:** Nasser Hajipour, Habib Allah Rashidzadeh, Jennifer Ketzis, Rouhollah Esmaeili seraji, Hamidreza Azizi, Iraj Karimi, Hossein Bagherniaee, Rohollah Montazeri

**Affiliations:** 1 Department of Pathobiology, Faculty of Veterinary Medicine, University of Tabriz, Tabriz 51666-16471, Iran; 2 Young Researchers and Elite Club, Najafabad Branch, Islamic Azad University, Najafabad 85141-43131, Iran; rashidzadehh@yahoo.com; 3 Department of Biomedical Sciences, Ross University School of Veterinary Medicine, Basseterre, St Kitts, West Indies; 4 Department of Clinical Science, Faculty of Veterinary Medicine, Shiraz University, Shiraz 71946-84471, Iran; r.e.seraji313@gmail.com; 5 Department of Pathobiology, Faculty of Veterinary Medicine, Shahrekord University, Shahrekord 64165-478, Iran; hr_azizi@yahoo.ca (H.A.); irkarimi@yahoo.com (I.K.); 6 University of Tabriz, Tabriz 51666-16471, Iran; h.bagerniaee87@ms.tabrizu.ac.ir; 7 Department of Food Hygiene and Quality Control, Faculty of Veterinary Medicine, Shahrekord branch, Islamic Azad University, Shahrekord 88179-33591, Iran; montazerivetmed@yahoo.com

**Keywords:** cysticercus ovis, *Taenia ovis*, sheep, goat, economic impact, seasonality

## Abstract

*Taenia ovis* larvae can result in economic losses in small ruminants due to condemnation of infected tissues or whole carcasses. From 2017 to 2018, the *T. ovis* prevalence in 16,180 sheep and 7560 goats at the Najafabad slaughterhouse in Isfahan was determined. More sheep (477; 2.9%) than goats (90; 1.2%) were found to be infected, and the prevalence was higher in animals <1 y (*p* < 0.0001), and higher in spring in sheep (8.2%) and goats (2.2%). Female sheep were more frequently infected than males (*p* < 0.0001); this did not hold true for goats. Of the tissues examined, *T. ovis* was found more often in the heart muscle of sheep compared with other tissues; however, infections in the heart muscle, masseter muscle, diaphragm, and triceps were similar in goats. Granulomas and caseous necrosis in the heart muscles were associated with the accumulation of mononuclear inflammatory cells and the formation of fibrous tissue around the parasite. Based solely on infected tissues found in this study, the economic loss caused by the presence of *T. ovis* larvae was estimated to be 4167 United States dollars (USD). Control methods, such as proper disposal of infected tissues and anthelmintic treatment of infected dogs, are necessary to decrease infection and prevent economic loss in small ruminants.

## 1. Introduction

Infection of small ruminants with the larva of the cestode, *Taenia ovis*, frequently referred to as *Cysticercus ovis* or ‘sheep measles’, occurs throughout much of the world, including in New Zealand, Australia, Canada, and some African countries, with more recent first reports of infection in Europe [1,2,3]. Dogs and, rarely, cats and foxes, serve as the definitive hosts with the adult cestode residing in the small intestine [4,5]. In the definitive host, gravid proglottids can be excreted daily, with over 80,000 eggs per proglottid per day infecting the environment [6]. Sheep, goats, and other small ruminants are infected during grazing by eating the egg containing an oncosphere. In the intestine, the oncosphere is released and, through blood circulation, reaches the liver, heart, lungs, spleen, muscles, and other organs, and develops into a cysticercus within three months [6,7]. These cysts are 6 to 100 mm in diameter and are oval, thin, fluid-filled, and contain a scolex [3,6]. The cysts remain viable for only a short period of time, approximately 6 weeks, after which the larva dies and the cyst becomes calcified. While small ruminants develop immunity to reinfection, the time period for this to develop depends on the immune status of the individual animal and, even after immunity, the calcified cysts can remain in the tissue for the remainder of the host’s life [8]. The definitive host, dogs, becomes infected by eating small ruminant viscera with live cysts; with growth of the adult cestode and production of gravid proglottids, the parasite life cycle is completed [7].

The infection of small ruminants with *T. ovis* is not considered clinically significant with no clinical signs in infected animals. However, although it is not a zoonotic parasite, the presence of viable or calcified cysts in meat and other organs of sheep and goats results in condemnation of the organs or even the entire carcass at post-slaughter inspection. According to the meat inspection guideline provided by the United Nations Food and Agriculture Organization (FAO), carcasses should be condemned if the infection is high, which is defined as two or more organs containing cysts [9]. If carcass infection is low or moderate, the FAO guideline recommends removing infected areas and storing the carcass at −10 °C for 10 days, to kill any cysts not removed during trimming, before going into the food chain [10]. The condemnation of whole carcasses and trimming of meat can result in large economic losses for the producers and the industry [5,11].

In Iran, few studies have been conducted on *T. ovis* prevalence in small ruminants, particularly goats [12]. However, small ruminants are a primary meat source and an important part of the agricultural economy in Iran [13]. Given the high economic impact of *T. ovis* infection in small ruminants, understanding the epidemiological aspects of the parasite, including determining the prevalence and risk factors, is important to facilitate control and treatment. Regional studies can assist in identifying climatic and production specific factors related to prevalence. The purpose of this study was to determine the prevalence of *T. ovis* infection in sheep and goats slaughtered at the Najafabad abattoir in Isfahan, the primary abattoir in the region, and assess factors that influence prevalence. Secondary objectives were to provide an estimate of the potential loss due to infections and confirm that the histopathology of the cysticerci were in agreement with previous reports.

## 2. Materials and Methods

This cross-sectional study was conducted on 23,740 small ruminants (7560 goats and 16,180 sheep) slaughtered at the Najafabad abattoir in Isfahan between 2017 and 2018. The Najafabad abattoir processes approximately 2000 sheep and 800 goats each month. The abattoir was visited at least eight days per month and a convenience sample from each day selected, based on the number that could be followed pre-slaughter through slaughter with equal numbers of animals examined per seasonal period. Before slaughter, the sex and age (<1 y or ≥1 y based on dentition), and general condition, posture, and behavior of each selected animal was recorded. After slaughter, the heart, diaphragm, thoracic and abdominal cavities, masseter muscles, triceps muscles, tongue, intestinal mucosa, liver, and spleen were examined for *T. ovis* cysticerci by cutting the organ/muscle in 3- to 4-mm sections as described by Heath and Lawrence [14]. An animal was considered infected if viable or calcified cysts were present. When found to be heavily infected, the organs and muscles were weighed.

All liquid or calcified cysts were placed in sterile containers with 10% formalin and transferred to the laboratory of the Faculty of Veterinary Medicine, Shahrekord University, for definitive diagnosis. Confirmation of *T. ovis* was based on morphology, specifically the size and appearance of the cyst, and the scolex, including the rostellar hooks [6,15]. For the histopathological study, infected tissues stored in 10% formalin were examined after the preparation of sections and staining with hematoxylin-eosin.

Prevalence was assessed based on all sheep and goats examined, season (spring: April, May, and June; summer: July, August, and September; autumn: October, November, and December; and winter: January, February, and March), age (<1 y and ≥1 y), and sex. Confidence intervals (95%) were calculated using the Clopper–Pearson exact binomial function on Epitools [16]. To determine if prevalence differed by season, age, or sex, a chi-square test was used. To estimate economic loss, the approximate weight of condemned, normally consumed, organs was determined and multiplied by the average value per kilogram (based on information from the Ministry of Agriculture and Union of Beef and Lamb) during the study period.

## 3. Results

Among 23,740 sheep and goats slaughtered, 567 (2.4%; 95% CI 2.2–2.6%) were infected with cysticerci of *T. ovis* (Table 1). The prevalence in sheep (2.9%) was significantly higher than in goats (1.2%) and significantly higher in female sheep than male sheep (*p* < 0.0001). In terms of age, infection in <1-year animals was significantly higher than ≥1-year animals (*p* < 0.0001). Seasonal analysis revealed significant differences (*p* < 0.0001) with prevalence in spring being the highest for both sheep and goats, and in summer the lowest. In regards to the tissues examined, the heart muscle was the most frequently infected tissue in sheep (Table 2). In goats, however, the heart muscle was infected as frequently as the masseter muscle, diaphragm, and triceps. Also, unlike the sheep, no cysticerci were found in the thigh muscle, liver, spleen, or intestinal mucosa of the goats.

**Figure 1:**
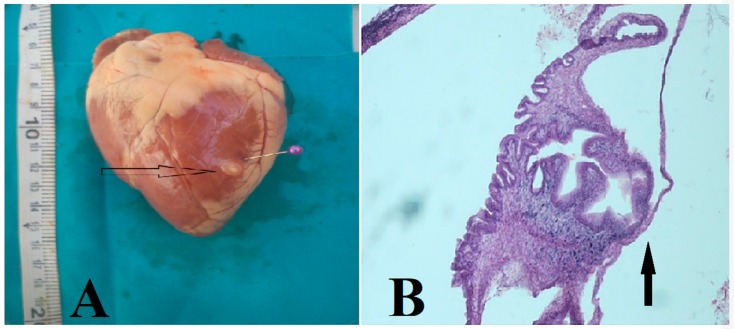
*Taenia ovis* cysticercus (**A**) and cross-section with protoscolex 10x magnification (H and E) (**B**) in cardiac muscle of sheep slaughtered in the Najafabad slaughterhouse, Isfahan, Iran.

**Figure 2:**
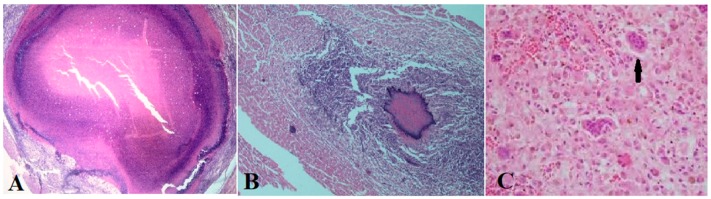
Caseous necrosis (**A**; H and E, 40x), granulomas (**B**; H and E, 10x), multinucleated giant cell (**C**; H and E, 1000x) in the cardiac muscle infected with *Taenia ovis* (A), and accumulation of mononuclear inflammatory cells and the formation of fibrous tissue around (H and E, 10x).

Viable cysts were fluid in nature, as previously described (Figure 1) [6,12] and degenerating cysts, based on the histopathology, consisted of granulomas and caseous necrosis with the accumulation of mononuclear inflammatory cells and the formation of fibrous tissue (Figure 2). This is also as previously described and is consistent across infected organs and tissues [6,12]. Histopathology results also were consistent with the morphological assessment and classification of the cysts as viable or degenerated.

Almost half of the infected heart muscles (250 of 533) and over half of the infected livers (90 of 143) and thigh muscles (140 of 234) during the study period were collected and destroyed due to the high infection intensity. The total weight of the heart and livers condemned was 220 kg with other condemned muscles examined during the study totaling 280 kg. At 1,000,000 RIAL/kg, loss was approximately 500,000,000 RIAL, equivalent to 4167 United States dollars (USD).

## 4. Discussion

In the present study, the prevalence of *T. ovis* in sheep slaughtered at the Najafabad abattoir, Isfahan was higher than the 1.3% found by Hashemnia et al. [12] in sheep slaughtered in Kermanshah and the 0.1% found by Oryan et al. [17] in sheep slaughtered in Fars Province. In comparison to other countries, the prevalence in sheep in the study presented here is similar to that reported for sheep in Tasmania (3.4%) and lower than that for eastern Ethiopia (26%) [2,18]. For goats, there is less prevalence data; however, the prevalence found in goats in this study is similar to that seen in local goats in Saudi Arabia (0.3–3.9%), but lower than that in imported goats in Saudi Arabia (1.7–5.3%) and goats in eastern Ethiopia (22%) [2,19]. The differences in the prevalence of *T. ovis* cysticerci infection in the present study, compared with other regions of Iran and other countries, should be interpreted with some caution, since there can be annual differences in prevalence [3], and most studies, including the one presented here, were conducted over only a few years. Differences in climate, particularly temperature, moisture, and rainfall, which impact survival of *T. ovis* environmental stages, also could provide an explanation for geographical and annual differences in prevalence [20]. This is particularly the case in comparing the results with those of Hashemnia et al. [12], whose study was conducted in a region with greater rainfall and lower average temperatures. Another factor that might contribute to prevalence differences is the population of carnivores, particularly stray dogs, in the grazing area of domestic ruminants and lack of proper efforts in segregating domestic and wild carnivores from livestock or their grazing areas. At the abattoir studied, the consistent destruction of infected tissues assists in breaking the life cycle. However, since inspection methods can lack sensitivity [14], inadvertently, infected meat can still be fed to dogs. To understand the contribution of domestic and wild canids to the prevalence in small ruminants, studies in domestic and wild canids in different regions are needed. Additionally, the frequency of anthelmintic administration, specifically of praziquantel, to domestic dogs used for herding sheep and goats, should be assessed to determine if targeted treatments could assist in breaking the life cycle.

Given the lack of sensitivity of standard meat inspection methods for *T. ovis*, identifying the most frequently infected animals and tissues could enable more thorough inspection of selected animals and tissues. In the study presented here, older animals were less frequently infected, which could be due to the animals’ immune system. While immunity is transmitted from ewes to lambs through colostrum, the immunity lasts only six to nine weeks, providing limited protection in younger animals [21]. However, this is somewhat in contrast to the finding of higher prevalence in female versus male sheep, since females often are slaughtered at an older age. This discrepancy might be attributed to a decrease in immunity during lambing, as occurs with other parasites. Classifying sheep at slaughter using more age categories (e.g., <1 y, 1–2 y, 2–3 y, etc.) could enable testing of age by sex interactions to assist in determining which age and sex groups to focus on at meat inspection.

The results of this study, with the heart muscle most frequently infected in sheep, are consistent with those of Hashemnia et al. [12]. However, while the study presented here did not find any cysticerci in several of the tissues inspected in goats, those that were infected have a similar frequency. Therefore, while the sheep heart muscle might be a better indicator of infection and potentially be inspected more closely than other tissues, goat meat inspection cannot as readily be focused.

The highest prevalence of infection in this investigation was in spring. This finding was in agreement with the results of Oryan et al. [22] in Fars province, Iran. Given that cysts, while not infective for several weeks, can be detected as soon as 10 d post-infection, prevalence at the abattoir can be reflective of recent as well as less recent exposure. In the region studied, the temperature and humidity in late spring and the easy access of animals to grass during this time likely play an important role in the epidemiology of the infection and the higher prevalence in the spring in the west of Iran [22]. This time of exposure also coincides with the time when lambs are grazing, since most lambs are born in early spring.

Histopathological studies revealed the presence of caseous necrosis in the heart muscle associated with the accumulation of mononuclear inflammatory cells and the formation of fibrous tissue around it. Hashemnia et al. [12] reported severe degenerative and necrotic changes in muscle fibers as well as cystic membrane entrapment by a fibroblast and inflammatory cell region in sheep infected with *T. ovis*. In chronic lesions, granuloma formation was observed around the parasite by the accumulation of epithelioid macrophages, multinucleated giant cells, lymphocytes, and many residues of necrotic cells [12].

Based on the findings of this study, *T. ovis* infection in sheep brought to the abattoir in Isfahan might not be as high as in other endemic areas. However, it still represents a large economic loss to sheep producers. The estimated loss presented in this study was based solely on the animals inspected by the researchers; hence, the total loss could be as much as three times higher if all animals were examined with the methods used in this study. Combining current inspection methods with focused inspections of certain tissues might increase the ability to identify infected animals, which could assist in breaking the life cycle. This could, however, increase the losses and the number of carcasses requiring freezing and storage. Treatment of dogs and small ruminants in spring, the time period for which transmission appears to be highest, could potentially impact prevalence. A cost-benefit analysis on the cost of praziquantel treatment of dogs compared with the economic losses at the abattoir could be used to demonstrate to farmers the benefit of control programs.

## Figures and Tables

**Table 1:** Prevalence of *Taenia ovis* cysticerci in sheep and goats, by age and sex, from the Najafabad abattoir, Isfahan, Iran, 2017–2018.

	Age		Sex		Season				Total
	<1 y	≥1 y	Female	Male	Spring	Summer	Autumn	Winter	
**Sheep**									
Sampled	10,960	5220	7600	8580	4045	4045	4045	4045	16,180
Positive	399	78	440	37	330	37	45	74	477
%	3.6 ^1^	1.5	5.8 ^1^	0.4	8.2 ^1^	0.9	1.1	1.8	2.9
95% CI%	3.3–4.0	1.2–1.9	5.3–6.3	0.3–0.6	7.3–9.0	0.6–1.3	0.8–1.5	1.4–2.3	2.7–3.2
**Goats**									
Sampled	2520	5040	5320	2240	1890	1890	1890	1890	7560
Positive	50	40	70	20	41	14	18	17	90
%	2.0 ^1^	0.8	1.3	0.9	2.2 ^1^	0.7	1.0	0.9	1.2
95% CI%	1.5–2.6	0.6–1.1	1.0–1.7	0.5–1.4	1.6–2.9	0.4–1.2	0.6–1.5	0.5–1.4	1.0–1.5

^1^ Prevalence was significantly different within factors assessed (age, sex, season) based on a chi-square test with *p*-values <0.0001.

**Table 2:** Prevalence of *Taenia ovis* cysticercosis in sheep (16,180) and goats (7560), by tissue, from the Najafabad abattoir, Isfahan, Iran, 2017–2018.

	Masseter Muscle	Heart Muscle	Diaphragm	Triceps	Intercostal Muscle	Thigh Muscle	Liver	Spleen	Intestinal Mucosa
**Sheep**									
Positive	368	450	380	350	289	234	143	98	199
%	2.3	2.8	2.3	2.2	1.8	1.4	0.9	0.6	1.2
95% CI%	2.1–2.5	2.5–3.0	2.1–2.6	1.9–2.4	1.6–2.0	1.3–1.6	0.7–1.0	0.5–0.7	1.1–1.4
**Goats**									
Positive	80	83	78	71	47	0	0	0	0
%	1.1	1.1	1.0	0.9	0.6				
95% CI%	0.8–1.3	0.9–1.4	0.8–1.3	0.7–1.2	0.5–0.8				
